# Two molecular measures of relatedness based on haplotype sharing

**DOI:** 10.1186/s12859-015-0802-y

**Published:** 2015-11-11

**Authors:** David Edwards

**Affiliations:** Centre for Quantitative Genetics and Genomics, Department of Molecular Biology and Genetics, Aarhus University, Blichers Allé 20, Tjele, 8830 DK Denmark

**Keywords:** Genomic relationship matrix, Multiset, Acyclic probabilistic finite automata, Haplotype sharing

## Abstract

**Background:**

Measuring the extent of shared ancestry between individuals or organisms is important in many fields, including forensic science, conservation genetics and animal breeding. The traditional approach is to calculate the expected degree of relatedness between individuals in a pedigree. This assumes that the founders of the pedigree are non-inbred and unrelated to each other, which is rarely the case. In contrast, molecular data allow measurement of actual relatedness without knowledge of a pedigree. Methods to do this have been proposed, but generally do not take the lengths of the genomic regions shared between individuals into account.

**Results:**

Two measures based on the extent of haplotype sharing between genomes are proposed. The intercept measure *B* estimates the fraction of shared genome between individuals, and the product measure *C* is closely related to the numerator relationship matrix *A*. Both are based on a model for the joint distribution of markers at the haplotype level. The two measures are compared to the pedigree-based measure *A* and to vanRaden’s *G*, a frequently used molecular measure, using a set of data comprising 5037 dairy cattle. The comparison criteria include the ability to capture genealogical relatedness and the prediction accuracy obtained when used in genomic prediction. Both *B* and *C* explain around 95 *%* of the variation in *A*, whereas *G* explains around 6 *%*. *G* captures genealogical relatedness poorly, particularly for distantly related individuals (second cousins or farther). Both *B* and *C* tend to be larger than *A* but this can be ascribed to the assumption of non-inbred unrelated founders. Using *C* in linear mixed models results in slightly higher prediction accuracy than *G*, and using *B* results in slightly lower prediction accuracy.

**Conclusions:**

The two proposed measures of relatedness capture genealogical relatedness well, outperforming vanRaden’s *G* in this respect. When used in genomic prediction models, the product measure leads to slightly improved prediction accuracy.

## Background

Estimating the extent of shared ancestry between individuals or organisms is central to many fields. Examples range from forensic science [1], studies of population structure [2, 3], and conservation genetics [4], to the mixed linear models used in genomic prediction and genome-wide association studies [5, 6].

Following Malêcot [7], measures of relatedness between two individuals are generally formulated in terms of the coefficient of coancestry, which is the probability that for a randomly selected gene, two alleles, one taken at random from each individual, descend from a single ancestral gene — that is, the probability that the alleles are identical-by-descent (IBD). Similarly, a measure of inbreeding for an individual is defined as the probability that the two alleles of a randomly selected gene are IBD [7]. When a pedigree is available, probabilities that two alleles are IBD from common ancestors within the pedigree can be calculated, and from these the classical measures of relatedness and inbreeding can be derived. The calculations typically assume that the founders are unrelated and not inbred, which is rarely the case. The measures depend on the choice of pedigree, and represent expected rather than realized relatedness and inbreeding, since they cannot incorporate the randomness inherent in meiosis.

With the advent of high-scale genotyping technologies such as single nucleotide polymorphism (SNP) arrays, it is possible to estimate realized relatedness directly from molecular data without knowledge of genealogy, and a variety of ways to do this are available [8, 9]. These generally take the form of genome-wise averages of single-SNP statistics, which have the disadvantage of not taking the lengths of genomic regions shared between two individuals into account [8]. In Section “Methods,” two novel measures based on the extent of haplotype sharing are described, and their properties studied. In Section “Results” the methods are applied to a set of data from dairy cattle, and compared to the classical pedigree-based measure and a measure due to vanRaden [10] that is widely used in animal breeding. Section “Software” describes the software used and Section “Discussion” gives a brief discussion.

## Methods

The methods developed in this paper are based on the following conceptual framework. The genome is divided into a series of physical intervals, and the variant DNA strings that may occur in the intervals are denoted *segments*. To each interval corresponds a set of segments that may occur in the interval, and these sets do not overlap: a segment that may occur in one interval may not occur in another. With some abuse of terminology one may identify the intervals with genes, and the possible segments with alleles. A specific genome is regarded as a collection of segments, and measures of relatedness between genomes are constructed in terms of similarity between such collections. For this the concept of a multiset is needed.

### Multisets

A multiset [11] is a generalization of the concept of a set that, unlike a set, allows multiple instances of its elements. The multiplicity of an element is the number of instances of the element in the multiset. For example, [2 figs, 5 pears, 3 plums ] is a multiset in which the element fig has multiplicity two. Note the use of square brackets [] to distinguish multisets from ordinary sets using curly brackets {}. A multiset corresponds to an ordinary set if the multiplicity of every element is one or zero.

Multiset intersection is a generalization of set intersection. The intersection of two multisets is formed by taking the minima of the multiplicities of the corresponding elements in the two multisets. For example:

[2 figs, 5 pears, 3 plums] ∩ [1 fig, 10 pears, 0 plums] = [1 fig, 5 pears, 0 plums].

The sum and product multiset operators, represented by + and × respectively, use the straightforward element-wise operations, for example:

[2 figs, 5 pears, 3 plums] + [1 fig, 10 pears, 0 plums] = [3 figs, 15 pears, 3 plums], and

[2 figs, 5 pears, 3 plums] × [1 fig, 10 pears, 0 plums] = [2 figs, 50 pears, 0 plums].

The cardinality of (number of elements in) a multiset *A* is written |*A*|. Some useful properties of the operators include the equations

$$\begin{array}{@{}rcl@{}} |A+B|&=&|A|+|B|, \\ A \times\, [B+C] &=& [A \times B] +\, [A \times C], \\ A +\, [B \cap C] &=& [A + B] \cap\, [A + C], \end{array} $$

that hold for any multisets *A*, *B* and *C* [11].

### Two measures of genomic relatedness

As described above a genome is taken to be composed of a collection of segments, taken from a larger pool of segments ${\mathcal {S}}$. Let *G*_*i*_ represent the genome of individual *i*, regarded as a collection of segments *s* in ${\mathcal {S}}$. Since there may be duplicates, *G*_*i*_ is a multiset. Let *x*_*is*_ represent the multiplicity of segment *s* in *G*_*i*_.

Let there be *p* intervals (loci). For $s \in {\mathcal {S}}$, let *l*(*s*)∈{1,…,*p*} indicate the interval associated with segment *s*. At each interval, an individual genome has two segments, corresponding to its two haplotypes. Thus each genome has in all 2*p* segments.

A natural definition of the similarity of two individuals is the fraction of genome that they share. So for individuals *i* and *j* the intersect measure of their similarity is defined as the cardinality of the intersection of the two genomes, divided by the total number of segments:

(1) $$\begin{array}{@{}rcl@{}} b_{ij} & = & |G_{i} \cap G_{j}|/2p  \\ & = & \sum_{s \in {\mathcal{S}}} x_{is} \wedge x_{js}/2p,  \end{array} $$

where *x*∧*y* is the minimum of *x* and *y*. Since |*G*_*i*_|=2*p*, we have *b*_*ii*_=1 for all *i*. For all *i* and *j*, 0≤*b*_*ij*_≤1. Also *b*_*ij*_=0 iff *G*_*i*_ and *G*_*j*_ have no common segments, and *b*_*ij*_=1 iff *G*_*i*_ and *G*_*j*_ are identical.

The product measure is defined similarly using the product operator:

(2) $$\begin{array}{@{}rcl@{}} c_{ij} & = & |G_{i} \times G_{j}|/2p  \\ & = & \sum_{s \in {\mathcal{S}}} x_{is} x_{js}/2p.  \end{array} $$

We have 0≤*c*_*ij*_≤2 for all *i* and *j*, with *c*_*ij*_=0 iff *G*_*i*_ and *G*_*j*_ have no common segments. In matrix terms *C*=(*c*_*ij*_) can be written as *C*=*X* *X* ^*T*^/2*p* where *X*=(*x*_*is*_) is the $N \times |{\mathcal {S}}|$ matrix of multiplicities.

To relate the two measures, note that when *x* and *y* take values in {0,1,2}, *xy* is given by , and *x*∧*y* by , so *x* *y*=2(*x*∧*y*)−*I*(*x*=*y*=1), where *I* is the indicator function. It follows that

(3) $$ c_{ij} = 2b_{ij} - \kappa_{ij},  $$

where *κ*_*ij*_, a measure of shared heterozygosity, is defined as

$$\kappa_{ij} = \#\{s \in {\mathcal{S}}: x_{is}=x_{js}=1\}/2p. $$

When *x*∈{0,1,2}, *x* ^2^=*x*+2*I*(*x*=2), so

$$\begin{array}{@{}rcl@{}} c_{ii} & = & \sum_{s \in {\mathcal{S}}} x_{is}^{2}/2p \\ & = & 1 + f_{i} \end{array} $$

where *f*_*i*_, a measure of homozygosity of individual *i*, is

$$f_{i}= \#\{s\in {\mathcal{S}}: x_{is}=2 \}/p. $$

To relate *C* to the numerator relationship matrix [12], let *θ*_*ij*_ be the coefficient of coancestry between individuals *i* and *j*, that is, the probability that for a randomly selected gene, two alleles, one taken at random from each individual, are IBD [7]. Define *𝜗*_*ij*_ similarly as the probability that for a randomly selected gene, two alleles, one taken at random from each individual, are identical, that is, identical-by-state (IBS). For *k*=1,…,*p*, write the two segments of individual *i* at interval *k* as $(s_{i1}^{k}, s_{i2}^{k})$, and similarly $(s_{j1}^{k}, s_{j2}^{k})$ for individual *j*. Then for *i*≠*j*,

$${\small{\begin{aligned} {}\sum_{s \in {\mathcal{S}}: l(s)=k} x_{is}x_{js} &= I\left(s_{i1}^{k}= s_{j1}^{k}\right) + I\left(s_{i1}^{k}= s_{j2}^{k}\right) + I\left(s_{i2}^{k}= s_{j1}^{k}\right) \\ &\quad+ I\left(s_{i2}^{k}= s_{j2}^{k}\right), \end{aligned}}} $$

so from (2)

(4) $$ \begin{aligned} {}2 p c_{ij} &= \sum_{k=1 \dots p} I\left(s_{i1}^{k}= s_{j1}^{k}\right) + I\left(s_{i1}^{k}= s_{j2}^{k}\right) + I\left(s_{i2}^{k}= s_{j1}^{k}\right)\\ &\quad+ I\left(s_{i2}^{k}= s_{j2}^{k}\right).  \end{aligned}  $$

Note that *𝜗*_*ij*_ is the probability of an event randomly chosen from the 4*p* identities on the right-hand side of Eq. (4). Hence *𝜗*_*ij*_=2*p* *c*_*ij*_/4*p*, and so *c*_*ij*_=2*𝜗*_*ij*_. Thus *c*_*ij*_ is twice the IBS-sense coefficient of coancestry *𝜗*_*ij*_.

Similarly, let *θ*_*i*_ be the coefficient of inbreeding for individual *i*, that is, the probability that for a randomly selected gene, the two alleles are IBD [7], and let *𝜗*_*i*_ be the corresponding IBS-sense quantity. When *i*=*j*, we obtain

(5) $$\begin{array}{*{20}l}  2 p c_{ii} & = \sum_{k=1 \dots p} I\left(s_{i1}^{k}= s_{i1}^{k}\right) + I\left(s_{i1}^{k}= s_{i2}^{k}\right) + I\left(s_{i2}^{k}= s_{i1}^{k}\right)\\ &\quad + I\left(s_{i2}^{k}= s_{i2}^{k}\right) \end{array} $$

(6) $$\begin{array}{*{20}l} &= \sum_{k=1 \dots p} 2 I\left(s_{i1}^{k}= s_{i1}^{k}\right) + 2 I\left(s_{i1}^{k}= s_{i2}^{k}\right) \end{array} $$

(7) $$\begin{array}{*{20}l} & = \sum_{k=1 \dots p} \left(2 + 2 I\left(s_{i1}^{k}= s_{i2}^{k}\right)\right) \end{array} $$

so *c*_*ii*_=1+*𝜗*_*i*_, and *f*_*i*_ is the IBS-sense coefficient of inbreeding *𝜗*_*i*_.

The additive, or numerator, relationship matrix is defined as *A*=(*a*_*ij*_) where

$$ a_{ij} = \left\{ \begin{array}{cc} 1+ \theta_{i} & \text{if} \ i=j \\ 2 \theta_{ij} & \text{otherwise} \\ \end{array} \right. $$

and as just shown

$$ c_{ij} = \left\{ \begin{array}{cc} 1+ \vartheta_{i} & \text{if} \ \text{i}=\text{j} \\ 2 \vartheta_{ij} & \text{otherwise} \\ \end{array} \right. $$

hence *C* and *A* are conceptually closely related. An assumption behind this assertion is discussed in Section “Discussion”.

### Defining the segmentation

To define the segmentation a statistical model in the form of an acyclic probabilistic finite automaton (APFA) [13] is used. Such models allow the extent of haplotype sharing within and between genomes to be quantified, and underlie the Beagle program [14, 15] that is widely used for processing high-dimensional SNP data. Phase estimation, imputation and model selection are performed simultaneously, using the algorithm described in [15]. Beagle is highly efficient, taking only a few minutes to process each chromosome for the data described in Section “Data and computations”, and performs well: for example, imputation accuracy rates generally exceed 97 *%* in cattle data [16].

**Fig. 1:**
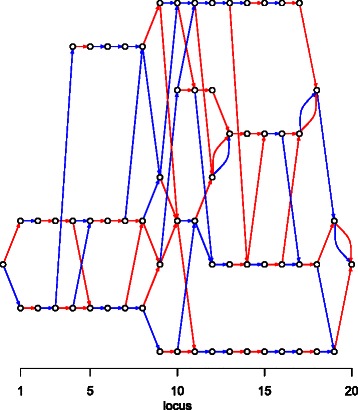
A simple APFA. An APFA ${\mathcal {A}}=(V,E)$ for *p*=20 markers. It has |*V*|=65 vertices and |*E*|=91 edges. The colour of the edges denotes the allele associated with the edge (red is “0” and blue is “1”). To each edge is attached a probability (not shown here)

An APFA is represented as a directed multigraph ${\mathcal {A}}=(V,E)$, where *V* is a vertex set and *E* an edge set: a small example is shown in Fig. 1. This is a model for the joint distribution of *p*=20 markers at the haplotype level. To each edge in *E* is attached a probability, such that the sum of probabilities of the outgoing edges from each vertex in *V* is one. Each haplotype corresponds to a path through the graph from the root (the leftmost vertex) to the sink (the rightmost vertex). The probability of a haplotype is the product of the probabilities of the edges in its root-to-sink path. See further [17, 18].

In [14, 15] the haplotypes that traverse a given edge are known as a haplotype cluster. Here a different perspective is adopted. The edges of the APFA are taken to represent chromosomal segments, that is, we set ${\mathcal {S}} = E$. So if two haplotypes traverse the same edge in an interval they are taken to share the same DNA in that interval, and if they traverse different edges, they are taken not to share DNA in that interval. The data may be represented as an *N*×|*E*| matrix *X* taking values in {0,1,2}, whose (*i,j*)th element specifies the multiplicity of segment *j* in individual *i*. The variables corresponding to the columns of *X* are called haplomarkers, and *X* is called the haplomarker design matrix.

**Fig. 2:**
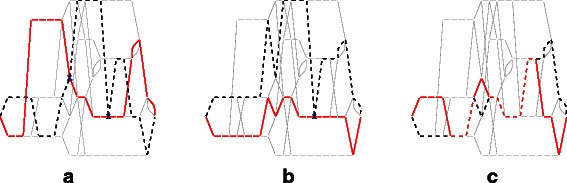
Recombination in an APFA. Maternal (**a**), paternal (**b**) and offspring genomes (**c**) are shown. In each genome, the maternal haplotypes are shown as red solid lines, and the paternal as black dashed lines. The overlap is shown as red dashed lines. Two crossovers occur in the maternal line and one in the paternal line: these are marked with “x”s

Figure 2 illustrates recombination under the model of Fig. 1. The two haplotypes of an individual correspond to two root-to-sink paths in the graph, and recombination is seen as crossing-over between the paths. If the individuals represented in Fig. 2 are taken in the order mother, father and offspring, we find the relationship matrices *B* and *C* to be $ B = \left (\begin {array}{ccc} 1.000 & 0.750 & 0.650 \\ 0.750 & 1.000 & 0.675 \\ 0.650 & 0.675 & 1.000 \\ \end {array} \right) $ and $ C = \left (\begin {array}{ccc} 1.000 & 0.750 & 0.875 \\ 0.750 & 1.000 & 0.900 \\ 0.875 & 0.900 & 1.450 \\ \end {array} \right) $. There are, for example, nine edges shared between the two haplotypes (red dashed lines) in the offspring genome, so the homozygosity of this individual is 0.45.

### Expected relatedness

Consider first three individuals, *i*, *j*, and *k*, where *i* and *j* are the parents of *k*. We examine how the parent-offspring relatedness measures depend on the relatedness of the parents. Specifically, expressions for the expectations of the parent-offspring relatedness conditional on the parental relatedness will be derived.

During meiosis the genomes *G*_*i*_ and *G*_*j*_ are first partitioned into two gametes, say $G_{i} = [{H_{i}^{1}} + {H_{i}^{2}}]$ and $G_{j} = [{H_{j}^{1}} + {H_{j}^{2}}]$ such that $|{H_{i}^{1}}|=|{H_{i}^{2}}|=|{H_{j}^{1}}|=|{H_{j}^{2}}|=p$. The partitioning process (segregation) is complex and stochastic, but only properties that are invariant to this are considered here. Then the genome $G_{k}= [H_{i}^{*} + H_{j}^{*}]$ is formed where $H_{i}^{*}$ is either ${H_{i}^{1}}$ or ${H_{i}^{2}}$, and $H_{j}^{*}$ is either ${H_{j}^{1}}$ or ${H_{j}^{2}}$, and the four combinations are equiprobable. Thus

$${\footnotesize{\begin{aligned} 4 E(|G_{i} \times G_{k}|) &= |G_{i} \times \left[{H_{i}^{1}} + {H_{j}^{1}}\right]| + |G_{i} \times \left[{H_{i}^{1}} + {H_{j}^{2}}\right]| \\ & \quad + |G_{i} \times \left[{H_{i}^{2}} + {H_{j}^{1}}\right]| + |G_{i} \times \left[{H_{i}^{2}} + {H_{j}^{2}}\right]| \\ & = |\left[G_{i} \times {H_{i}^{1}}\right] + \left[G_{i} \times {H_{j}^{1}}\right] + \left[G_{i} \times {H_{i}^{1}}\right] + \left[G_{i} \times {H_{j}^{2}}\right] \\ & \quad+ \left[G_{i} \times {H_{i}^{2}}\right] + \left[G_{i} \times {H_{j}^{1}}\right] + \left[G_{i} \times {H_{i}^{2}}\right] + \left[G_{i} \times {H_{j}^{2}}\right]| \\ & = 2|G_{i} \times G_{i}| + 2 |G_{i} \times G_{j}| \end{aligned}}} $$

so

(8) $$\begin{array}{@{}rcl@{}}  E(c_{ik}) & = & (c_{ii} + c_{ij})/2. \end{array} $$

Similarly

(9) $$ 4 E(G_{k} \times G_{k}) = 8 p + 2 |G_{i} \times G_{j}|  $$

so

(10) $$ E(c_{kk}) = 1+ c_{ij}/2   $$

and

(11) $$ E(f_{k}) = c_{ij}/2.   $$

Now consider four individuals, *h*, *i*, *j* and *k*, where again *i* and *j* are the parents of *k*, and where the relatedness between *h*, *i* and *j* is known.

$${\small{\begin{aligned} 4 E(|G_{k} \times G_{h}|) &= |[{H_{i}^{1}} + {H_{j}^{1}}] \times G_{h}| + |[{H_{i}^{1}} + {H_{j}^{2}}] \times G_{h}| \\ & \quad+ |[{H_{i}^{2}} + {H_{j}^{1}}] \times G_{h}| + |[{H_{i}^{2}} + {H_{j}^{2}}] \times G_{h}| \\ & = 2 |G_{i} \times G_{h}| + 2 |G_{j} \times G_{h}|, \end{aligned}}} $$

so

(12) $$  E(c_{kh})=(c_{ih}+c_{jh})/2.  $$

An expression for the expectation of the intersect measure may be derived in a similar fashion:

(13) $$  E(b_{ik})= E(b_{jk})= 1/2 + c_{ij}/4.  $$

but I have not been able to derive an expression for *E*(*b*_*kh*_) corresponding to (12).

Expressions (8), (??) and (12) are identical to those used in the calculation of the numerator relationship matrix using the algorithm of [19]. When only a subset of individuals in a pedigree are genotyped, a hybrid expected/realized relationship matrix *R*=(*r*_*ij*_) exploiting both pedigree and genomic information can be obtained using the following simple modification to the algorithm.

Order the individuals so that parents precede their offspring and label them 1,…,*N*. Write the subset of genotyped individuals as *S*, and for all *i,j*∈*S*, set *r*_*ij*_=*c*_*ij*_, the realized genomic relatedness described above. For *k*=1,…*N*, derive the relatedness between an individual *k* and the preceding individuals as follows. When *k*∉*S*, set

$$\begin{array}{@{}rcl@{}} r_{kk} = 1 + r_{ij}/2 \end{array} $$

where *i* and *j* are the parents of *k*. If either or both *i* and *j* are unknown (i.e., not in the pedigree) assume they are unrelated, that is, use *r*_*ij*_=0 in this calculation. For each *h*∈{1,…,*k*−1}, if {*h,k*}⫅̸*S*, set

$$\begin{array}{@{}rcl@{}} r_{hk} = r_{kh}= (r_{ih} + r_{jh})/2, \end{array} $$

where again *i* and *j* are the parents of *k*. If *i* is unknown, use *r*_*ih*_=0, and if *j* is unknown, use *r*_*jh*_=0 in this calculation.

This algorithm adjusts the expected relationships downstream of *S* in the pedigree. An alternative method that adjusts all the relationships outside of *S* is sketched in the following subsection.

### Modifying *A*

This subsection describes an established technique that is useful in various contexts. The individuals are partitioned into two groups, so that  and . We regard *A* as a covariance matrix and wish to construct a new $\tilde {A}$ for which the marginal covariance of group one is set to $A_{11}^{*}$, and the conditional (co)variance of group two, given group one, is kept the same. That is to say, such that *A* ^12^, *A* ^21^=(*A* ^12^)^*T*^ and *A* ^22^ are retained. So we require that  where ∗ denotes unspecified and *E* is an increment matrix to be found. Using standard results on inverses of partitioned matrices we obtain

$$A_{11}^{*} = \left(A^{11}+E + A^{12}\left(A^{22}\right)^{-1}A^{21}\right)^{-1} $$

and so

$$\begin{array}{@{}rcl@{}} E &=& \left(A_{11}^{*}\right)^{-1} - \left(A^{11} + A^{12}(A^{22})^{-1}A^{21}\right) \\ &=& \left(A_{11}^{*}\right)^{-1} - \left(A_{11}\right)^{-1} \end{array} $$

is the required increment. Write the matrix obtained in this way $\tilde {A} = A|A_{11}^{*}$. This technique is used when a subset of individuals in a pedigree are genotyped, to compute a hybrid expected/realized relationship matrix exploiting both pedigree and genomic information [20, 21].

## Results

In this section empirical comparisons are made between the proposed relationship matrices *B* and *C*, the numerator relationship matrix *A* derived from the pedigree, and the matrix *G* of vanRaden [10].

### Data and computations

The data used in this analysis are genotypes and complex traits for 5037 Nordic Holstein bulls. The 5037 bulls were genotyped using a 50K chip and then imputed with Impute2 [22] to 777K (HD), using a reference panel of 1197 HD genotyped bulls. Five traits are examined below: protein, fat, yield, body and mastitis. These are de-regressed proofs (DRP) derived from genetic evaluations in December 2013. A detailed description of their definitions and derivations is available from the Danish Agricultural Advisory Centre (https://www.landbrugsinfo.dk). The year of birth of the bulls ranged from 1974 to 2009. The 3914 bulls born until 2004 were taken to comprise the training set, and the remaining 1394 bulls born from 2005 to 2009 were taken to comprise the test set.

The pedigree-based relationship matrix (*A*) for the 5037 bulls was derived from the Nordic Holstein pedigree of year 2013 (which contains a total of 134832 animals) in the standard way.

The genomic relationship matrix (*G*) following [10] was calculated from the marker data, using

(14) $$ g_{ij}= \frac{\sum_{k=1 \dots p} (m_{ik} - \bar{m}_{k})(m_{jk} - \bar{m}_{k}) }{\sum_{k=1 \dots p} 2 \bar{m}_{k}(1-\bar{m}_{k}) }   $$

where *M*=(*m*_*ik*_) is the *N*×*p* marker design matrix whose elements take values in {0,1,2}, and $\bar {m}_{k}$ is the mean allele frequency of the relevant allele of the *k*th marker, that is, $\bar {m}_{k}= \sum _{i=1 \dots N} m_{\textit {ik}}/N$. Thus the allele frequencies are set to those in the current sample.

Beagle version 3.3.2 was applied to the unphased marker data from the Holstein bulls, and the *B* and *C* matrices were derived from the Beagle output files. Beagle uses two tuning parameters, *m* and *b*. The larger the parameters, the simpler the selected APFA. The settings *m*=1 and *b*=0, suggested in [15, 18], were used below in the following sections, except Section “Prediction”: here the settings *m*=4 and *b*=0.2 suggested in [23] were used, since they result in slightly better prediction accuracy.

### Relatedness and pedigree distance

In Sections “Two measures of genomic relatedness” and “Expected relatedness” it was seen that there is a close conceptual relationship between *C* and the numerator relationship matrix *A*. This section examines empirically the extent to which the measures capture the genealogical relationships between individuals. This is done in several ways.

**Fig. 3:**
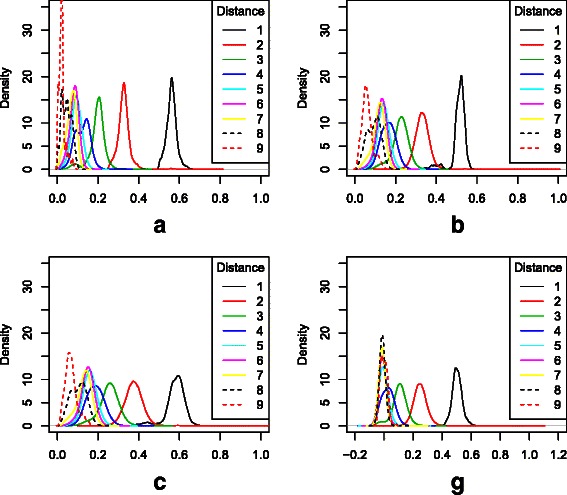
Sample densities of relationship measures by pedigree distance. Sample densities broken down by pedigree distance for four measures: **a** the numerator relationship matrix; **b** the intersect measure; **c** the product measure and **g** the genomic relationship measure of [10]

In a crude but informative approach, the distance between each pair of bulls may be calculated as the length of the shortest path between the bulls in the pedigree. For example, full- and half-sibs are at distance two, first cousins are at distance four, and second cousins are at distance six. Figure 3 shows sample densities of the four measures broken down by distance for all pairs of animals. Corresponding summary statistics are shown in Table 1.

**Table 1:** Summary statistics of relationship measures broken down by distance

	A		B		C		G	
Distance	mean	sd	mean	sd	mean	sd	mean	sd
1	0.562	0.026	0.510	0.037	0.579	0.048	0.504	0.035
2	0.328	0.035	0.332	0.040	0.377	0.049	0.252	0.052
3	0.195	0.042	0.223	0.042	0.253	0.050	0.104	0.054
4	0.122	0.037	0.163	0.037	0.186	0.044	0.025	0.046
5	0.094	0.027	0.140	0.030	0.160	0.036	-0.002	0.033
6	0.082	0.024	0.130	0.029	0.148	0.034	-0.010	0.027
7	0.068	0.025	0.117	0.031	0.132	0.037	-0.012	0.024
8	0.046	0.023	0.092	0.034	0.103	0.039	-0.011	0.021
9	0.023	0.016	0.061	0.026	0.067	0.029	0.000	0.024

The parent-offspring pairs are clearly identified by all four measures, but for the more distant pairs there appears to be least separation for the *G* measure. For the *A* measure, distances of four and above are less than 0.2, with a spike close to zero at distance 9, reflecting the assumption of unrelated founders. There are distinct peaks for most distances, indicating good separation between these. The *B* and *C* measures at distance four and above are larger, being less than 0.3, also with good separation. The *G* measures for distance five and above are centered around zero, so about half of the values are negative, and there is poor separation. This suggests that *G* performs relatively poorly for distantly related individuals, say second cousins or farther.

### Comparison with pedigree-based relationships

**Fig. 4:**
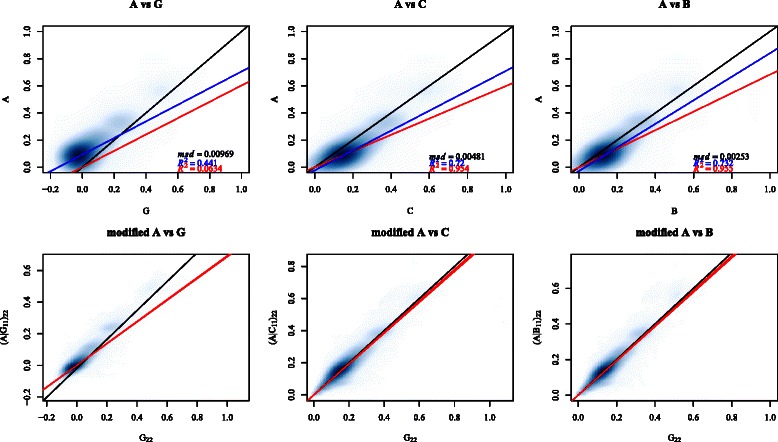
Comparison of relationship coefficients of *A* with those of *G*, *C* and *B*. The three upper subplots show smoothed scatter plots of the off-diagonals of A with those of G, B, and C. Identity lines are shown in black, and linear regression lines with and without intercept terms are shown in blue and red, respectively. The mean squared deviation (average of $\sum _{i \neq j} (x_{\textit {ij}}-y_{\textit {ij}})^{2}$) and the adjusted *R* ^2^ values corresponding to the regressions are also shown. The three lower subplots display the consistency of *G*, *C* and *B* with *A*, showing smoothed scatter plots of (*A*|*G*_11_)_22_ versus *G*_22_, (*A*|*C*_11_)_22_ versus *C*_22_ and (*A*|*B*_11_)_22_ versus *B*_22_, respectively. Identity lines are shown in black, and linear regression lines in red: these are overlaid from 10 replicates

The distance measure just described is crude since pairs of animals at a given distance may be more or less related, due to varying numbers and lengths of lineage paths between them and common ancestors in the pedigree. The *A* matrix takes this into account and is the natural pedigree-based measure. The upper three subplots of Fig. 4 show smoothed scatterplots of *A* versus *G*, *B*, and *C*, for all distinct pairs of animals in the data. It is seen that the bulk of the points lie above the identity line in the *A* versus *G* plot, and under the line in the *A* versus *B*, and *A* versus *C* plots: that is, *G* tends to underestimate *A* whereas *B* and *C* tend to overestimate *A*. See also Table 1. Adjusted *R* ^2^ statistics based on simple linear regression models with no intercept, as shown in Fig. 4, indicate that *G* only explains around 6 *%* of the variation of *A*, whereas both *B* and *C* explain around 95 *%*.

### Comparison using consistency

The tendency for *B* and *C* to be larger than *A* could be due to the assumption of non-inbred, unrelated founders that underlies *A*: if this is false, deflated estimates of relatedness and inbreeding would result. To examine this possibility we use the technique described in Section “Modifying *A*” to examine the consistency of *B*, *C* and *G* with *A*, in the following way. A random sample of 1000 animals from the genotyped Holstein bulls is taken, and called group one. The matrices *A*|*G*_11_, *A*|*B*_11_ and *A*|*C*_11_ are derived and compared with the realized relationships, that is, the off-diagonals of (*A*|*G*_11_)_22_ are compared with those of *G*_22_ and so forth. The process is repeated for 10 random samples of size 1000. The three lower subplots in Fig. 4 show the results. It is seen that *B* is highly consistent with *A*: the realized relatedness measures in *B*_22_ are very close to the adjusted values (*A*|*B*_11_)_22_. The same is true of *C*. But *G* shows poor consistency with *A*: the realized relatedness measures in *G*_22_ tend to exceed the adjusted values (*A*|*G*_11_)_22_.

### Comparison of inbreeding coefficients

**Fig. 5:**
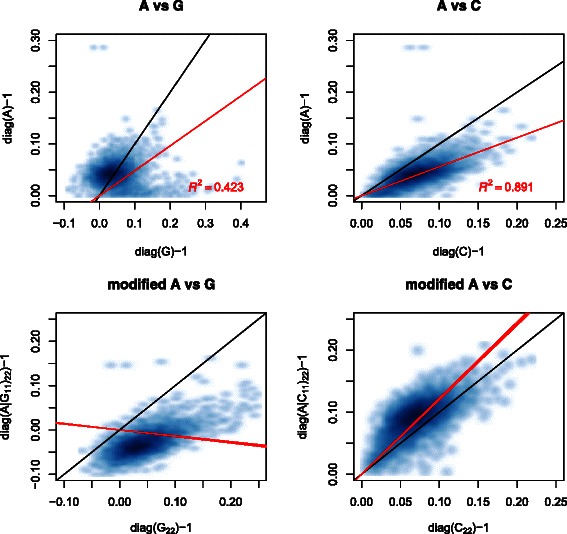
Comparison of inbreeding coefficients from *A* with those from *G* and *C*. The two upper subplots show smoothed scatter plots comparing the inbreeding coefficients from *A* and *G*, and from *A* and *C*, respectively. Identity lines are shown in black and linear regression lines (with zero intercept) in red. The corresponding adjusted *R* ^2^ values are also shown. The two lower subplots display the consistency of the estimates of inbreeding coefficients from *G* and *C*, respectively, with those from *A*. Smoothed scatter plots of inbreeding coefficients from (*A*|*G*_11_)_22_ versus *G*_22_, and (*A*|*C*_11_)_22_ versus *C*_22_ are shown. Identity lines are shown in black, and linear regression lines (with zero intercept) in red: these are overlaid from 10 replicates

Finally, Fig. 5 shows smoothed scatter plots comparing the inbreeding coefficients obtained from *A* with those from *G* and *C*. It is seen that *C* explains 89 *%* of the variation in *A* whereas *G* explains only 42 *%*. The inbreeding coefficients from *A* are non-negative, but negative values occur in *G*. The inbreeding coefficients from *C* are consistently larger than those from *A*, which may reflect assumptions of non-inbred unrelated founders underlying *A*. To examine this, the consistency of the inbreeding coefficients from *C* and *G* are compared using the method just described: the results are shown in Fig. 5. The coefficients from *C*_22_ are slightly smaller than those from (*A*|*C*_11_)_22_. Thus the difference may at least in part be due to assumptions of non-inbred unrelated founders. The consistency of the inbreeding coefficients from *G* with those from *A* is very poor. Estimates of inbreeding coefficients based on *G* may be sensitive to choice of allele frequencies in the base population [10].

### Prediction

To compare the use of the relatedness measures in prediction, breeding values were predicted using a genomic restricted maximum likelihood (G-REML) model of the form

(15) $$ y = \textbf{1} \mu + g + e   $$

where *y* is the response vector, *μ* is the overall mean, **1** is a vector of 1’s, *g* is a vector of breeding values, and *e* is a vector of residuals. It is assumed that $g \sim N(0, V {\sigma ^{2}_{g}})$ and independently $e \sim N(0, D {\sigma ^{2}_{e}})$, where *V* is a relationship matrix (i.e. *A*, *B*, *C*, or *G*), and *D* is a diagonal matrix with elements $d_{\textit {kk}}=(1 - {r_{k}^{2}})/{r_{k}^{2}}$ to account for heterogeneous residual variances due to varying reliability ${r_{k}^{2}}$ of the complex trait *y*.

The analysis was performed with the package DMU [24] applied to data from the training set, using the four relationship matrices. To examine prediction using less related individuals, a reduced training set was constructed by excluding all sires and grandsires of any animal in the test set from the training set. The prediction accuracy, that is, the correlation between the predicted and observed values in the test set, are shown in Table 2. It is seen that *C* consistently has the highest prediction accuracy, though the improvement over *G* is modest, of the order of 0.4 *%* when the full training set is used, and 0.6 *%* when closely related animals are excluded from the training set. In contrast, *B* has slightly less prediction accuracy than *C* for both the full and the reduced training set.

**Table 2:** Prediction accuracy (correlation) for G-REML using the four relationship matrices

	Full training set				Reduced training set			
Trait	A	B	C	G	A	B	C	G
protein	0.498	0.661	0.670	0.667	0.219	0.556	0.562	0.559
fat	0.474	0.625	0.651	0.643	0.220	0.526	0.556	0.549
body	0.490	0.567	0.570	0.565	0.377	0.518	0.526	0.512
mast	0.454	0.572	0.585	0.581	0.298	0.504	0.513	0.514
yield	0.513	0.656	0.667	0.663	0.203	0.543	0.554	0.549

## Software

Beagle version 3.3.2 was used to select APFA on which the measures are based. A C++ program, available from the author, was written to construct the *B* and *C* relationship matrices from Beagle output files. DMU [24] was used to perform the REML analyses. The remaining computations were performed using R: in particular, the *A* matrix was computed using the pedigree package, and Figs. 1 and 2 were constructed using the gRapfa package [18].

## Discussion

Two novel measures of relatedness based on shared haplotypes were introduced in Section “Methods”. The intersect measure (*B*) is an estimate of the fraction of shared genome, and the product measure (*C*) is closely related to the numerator relationship matrix (*A*) [12].

The framework underlying the measures is that of a diploid genome divided into intervals and segments (or genes and alleles) in which it is assumed that segments are not shared between intervals, so that the multiplicity of each segment in a genome is in {0,1,2}. It would be interesting to examine whether the measures can be extended to polyploid genomes, and whether the assumption of no shared segments, which cannot accommodate phenomena such as gene duplication, can be relaxed.

The close conceptual relation between *C* and *A* rests implicitly on an assumption that whenever two haplotypes share the same segment (in the APFA context, traverse the same edge) their chromosomal segments are indeed identical (IBS). This is a strong assumption, and most likely only approximately true. Probably the reason that the proposed measures capture genealogical relatedness well here is that the APFA-based segmentation is in reasonable accordance with this assumption. If the sample size were small, a overly simple APFA would be selected, leading to over-estimation of haplotype sharing. Similarly, if segments were defined directly using the marker alleles (say, with two segments per interlocus interval corresponding to the alleles of a flanking marker), the assumption would be violated, and the resulting measures may be expected to capture relatedness poorly. If full-sequence data are available, it would in principle be possible to verify the assumption for the APFA-based segmentation, or perhaps to develop an improved segmentation method.

In Section “Results” it was shown that the tendency for *B* and *C* to be larger than *A* can be ascribed to the assumptions of non-inbred, unrelated founders that underlie *A*. An alternative explanation could be that the segmentation method chosen here tends to overestimate the extent of haplotype sharing. Further research into this would be useful.

A comparison of the prediction accuracy in a mixed linear model using the relationship matrices as covariances found that *C* performed consistently better than *G*, with an improvement of about 0.4 *%* when all available animals were used in the training set, increasing to 0.6 *%* when close relatives were excluded. There is intense interest in methods to improve prediction accuracy in genomic selection programmes [25], since small improvements may represent substantial economic gains for the breeding company, and the present methods may contribute to this goal.

Note that as described above *C* takes the form *X* *X* ^*T*^/2*p*, where *X* is the *N*×|*E*| haplomarker design matrix. Hence the use of *C* in (15) is equivalent to the model *y*=**1** *μ*+*X* *h*+*e* with random haplomarker effects $h \sim N(0, I_{|E|} {\sigma _{g}^{2}})$ and independent error $e \sim N\left (0, D {\sigma _{e}^{2}}\right)$. From Eq. (14), *G* takes the form (*M*−*δ*)(*M*−*δ*)^*T*^/*η* where *M* is the *N*×*p* marker design matrix, and *δ* and *η* are shift and scale constants. Although different shift and scale transformations of the (haplo)marker variables would lead to different relationship matrices, they would not affect the predictive ability of the models [26]. So in this sense the comparison between *C* and *G* in the prediction context is between the predictive power of *X* and *M*, rather than between the relatedness measures *per se*.

It is straightforward to construct weighted versions of the measures. Let *w*_1_,…,*w*_*p*_ be a set of apriori given non-negative numbers such that $\sum _{i=1 \dots p} w_{i}= 1$. These could for example be proportional to inter-marker distances, or to probabilities of the existence of a quantitative trait locus (QTL) in the respective interval in order to quantify trait-specific relatedness. Expressions (1) and (2) are replaced by $\sum _{s \in {\mathcal {S}}} w_{l(s)} (x_{\textit {is}} \wedge x_{\textit {js}})$ and $\sum _{s \in {\mathcal {S}}} w_{l(s)} x_{\textit {is}} x_{\textit {js}}$, respectively.

The present methods have a certain similarity of approach to that of the Chromopainter program [27]. This seeks to explore admixture in SNP data sampled from multiple populations. Given SNP data for a set of recipient chromosomes, and for a set of donor chromosomes, it forms each recipient chromosome as a mosaic of donor chromosomes, by applying the haplotype copying model [28] in a hidden Markov model framework. This has been used to explore human migratory history [29]. The present methods provide an alternative modelling approach in which it is not necessary to prescribe a donor/recipient ordering. A review of genomic similarity measures from the population structure perspective is given in [30].

A natural way to display patterns of relatedness is to apply principal coordinates analysis ([31], Chapter 14), using − log(*b*_*ij*_) as a distance measure between individuals *i* and *j*. Also the length of shared regions is informative: on average, the longer the shared regions, the more recent the ancestor(s). The location of the shared regions may sometimes also be of interest, for example, when there is knowledge of the location of genetic variants influencing a complex trait.

## Conclusions

Two novel molecular measures of relatedness based on haplotype sharing are described. The intersect measure estimates the fraction of shared genome between individuals, and the product measure has a close conceptual relationship with the coefficient of coancestry. Both capture genealogical relatedness well, outperforming vanRaden’s *G* in this respect. When used in genomic prediction models, the product measure leads to slightly improved prediction accuracy.

## Availability of data and materials

The cattle data are the property of the Danish Cattle Federation (Aarhus, Denmark), Faba Co-op (Helsinki, Finland), Seges (Aarhus, Denmark), Växa Sverige (Uppsala, Sweden), Swedish Dairy Association (Stockholm, Sweden), and Nordic Cattle Genetic Evaluation (Aarhus, Denmark), and are not publicly available.
